# The interplay between NLRP3 inflammasome and metabolic signals in gouty arthritis

**DOI:** 10.3389/fimmu.2026.1761121

**Published:** 2026-03-12

**Authors:** Dongyi Cao, Hangyi Pu, Xiaolin Yuan, Zhengyan Li, Xiaoling Yu, Xiaoke Shi

**Affiliations:** 1 Department of Pharmacy, Kunming Municipal Hospital of Traditional Chinese Medicine, Kunming, China; 2 Chengdu Institute of Biology, Chinese Academy of Sciences, Chengdu, China; 3 Innovative Institute of Chinese Medicine and Pharmacy, Chengdu University of Traditional Chinese Medicine, Chengdu, China

**Keywords:** gouty arthritis, metabolic signals, monosodium urate crystals, NLRP3 inflammasome, therapeutic strategy

## Abstract

The pathogenesis of gouty arthritis (GA) begins with the deposition of monosodium urate (MSU) crystals in the joints. This crystal deposition triggers a critical inflammatory response by activating the NLRP3 inflammasome, which in turn drives the maturation and release of pro-inflammatory cytokines such as IL-1β. Beyond this well-defined inflammatory axis, metabolic dysregulation is increasingly recognized as a core component of GA pathogenesis. This paper systematically reviews the crosstalk between metabolic signaling and the NLRP3 inflammasome in GA, elucidating how MSU crystals serve as a bridge between hyperuricemia (HUA) and innate immune activation. Furthermore, we elaborate the dual role of metabolic factors: acting both as “primer” and “amplifiers” of NLRP3 inflammasome activation, significantly lowering its activation threshold. This mechanistic association offers novel therapeutic insights for GA management: synergistic regulation of metabolic signaling alongside targeted inhibition of NLRP3 inflammasome activation enables more effective therapeutic interventions. Defining gout as a “metabolic-inflammatory” disorder has led to the development of novel dual-target therapeutic strategies—simultaneously alleviating inflammatory symptoms while regulating metabolic abnormalities. Such approaches hold significant promise for effectively preventing and controlling gout attacks, whilst reducing the risk of long-term complications.

## 1 Introduction

Gouty arthritis (GA), the most prevalent inflammatory arthropathy worldwide, manifests as excruciatingly painful flares characterized by erythema, swelling, and localized heat in peripheral joints—most commonly the first metatarsophalangeal joint (1). Beyond its acute presentation, untreated GA progresses to chronic joint destruction and tophi formation, imposing substantial socioeconomic burdens (2). According to the Global Burden of Disease Study (GBD), worldwide GA cases reached 55.8 million by 2020—a 22.5% increase since 1990—and are projected to approach 95.8 million by 2050 (3). In addition to the common risk factors of old age and male gender (1, 4), nutritional excesses such as alcohol consumption, high-purine meats, seafood, and fructose-containing sodas are also risk factors for GA, as they promote hyperuricemia (HUA) and trigger acute flares by elevating uric acid burden (5).

HUA represents a primary etiological driver of GA pathogenesis, and defined as serum urate concentrations above the normal range (0.42 mmol/L or 7 mg/dL) (2). However, emerging evidence confirms that only up to 36% of hyperuricemic individuals develop clinical GA, indicating that HUA alone is insufficient to induce GA (6). The critical transition occurs when elevated serum urate drives the formation and deposition of monosodium urate (MSU) crystals within articular and periarticular tissues. This MSU crystal deposition represents the essential pathogenic event preceding clinically evident gout. Deposited MSU crystals function as damage-associated molecular patterns (DAMPs), recognized by innate immune cells (e.g., macrophages) via pattern recognition receptors (PRRs), including Toll-like receptors (TLRs) and NOD-like receptors (NLRs). Critically, MSU engagement triggers canonical NLRP3 inflammasome activation in macrophages and monocytes (7), which is the central driver of acute GA flares. *In vitro* studies indicate that MSU-induced interleukin-1β (IL-1β) release is primarily mediated by NOD-like receptor family pyrin domain containing 3 (NLRP3) inflammasome activation: MSU recognition triggers NLRP3 inflammasome assembly, resulting in caspase-1-dependent cleavage and release of bioactive IL-1β. This cytokine amplifies local inflammation and recruits/activates inflammatory leukocytes (8). Ultimately, these mechanisms underlie tophus formation and sustain GA pathogenesis.

GA itself is often associated with metabolic syndrome such as obesity, insulin resistance, dyslipidemia, hypertension, and type 2 diabetes (9). Beyond simply providing the substrate for MSU crystal formation, emerging evidence highlights that these metabolic disturbances profoundly influence NLRP3 inflammasome activity (10). Metabolites like saturated fatty acids (SFAs), cholesterol crystals, elevated glucose, and mitochondrial dysfunction associated with metabolic stress can act as priming signals, enhance activation of NLRP3 inflammasome, or directly modulate inflammasome components (11). Conversely, NLRP3 activation and chronic IL-1β release can exacerbate metabolic dysfunction (12), creating a potential feed-forward loop that perpetuates both inflammation and metabolic disease.

This review will synthesize current research on how diverse metabolic factors, including lipids, glucose, mitochondrial metabolites, adipokines, and dietary components, interact with and regulate the NLRP3 inflammasome. It further explores how this regulation contributes to the complex bidirectional interplay between the NLRP3 inflammasome and metabolic signals, representing a new entry point for understanding GA pathogenesis.

## 2 MSU crystals: a bridge linking metabolic dysregulation and innate immunity

The pathogenesis of GA critically hinges on the formation and deposition of MSU crystals within joints and periarticular tissues. These crystalline structures are not merely byproducts of HUA (elevated serum uric acid levels exceeding its solubility limit), they also serve as an important pathological bridge between underlying metabolic dysregulation and activation of the innate immune system.

### 2.1 Metabolic dysregulation fuels crystal precursors

Uric acid (UA), a weak organic acid, circulates predominantly as the monoanion urate under physiological conditions (pH 7.4, 37°C) (13). As the terminal metabolite of exogenous and endogenous purine degradation, UA is generated through a series of enzymatic reactions, with xanthine oxidase (XO) serving as the rate-limiting enzyme in the final step of urate production (14). Elevated serum UA levels, resulting from an imbalance between production and renal excretion (15), define HUA when exceeding a specific threshold. This hyperuricemic state initiates MSU crystal deposition; however, not all HUA progresses to crystal formation.

HUA arises from two core mechanisms: abnormal purine metabolism (leading to UA overproduction) and dysfunction of urate transporters (leading to impaired UA excretion) (16). Multiple metabolically driven pathways converge to fuel UA overproduction. Dietary and substrate overload — notably excessive intake of purine-rich foods, fructose (via accelerated ATP degradation), and alcohol — directly saturate the purine catabolic pathway, significantly amplifying UA generation through XO activity (17). Cellular metabolic stress has also been found to increase UA production: insulin resistance (18), hypertension-induced renal vasoconstriction (19), and accelerated cellular turnover (e.g., myeloproliferative disorders) increase substrate availability (nucleic acids, ATP) (17) for UA synthesis. In addition, rare genetic causes such as hypoxanthine-guanine phosphoribosyltransferase (HPRT) deficiency and phosphoribosyl pyrophosphate synthetase (PRS) hyperactivity constitutively drive pathological UA overproduction (14).

Under physiological conditions, the kidneys account for the majority of daily UA clearance (20), renal hypoplasia leads to secondary elevated serum UA. Three major urate transporters, including URAT1 (SLC22A12), GLUT9 (SLC2A9), and ABCG2 (BCRP), are critical regulators of serum UA homeostasis, with their dysfunction directly linked to urate transport disorders (21). URAT1, encoded by the SLC22A12 gene, serves as the dominant apical urate reabsorption exchanger in the proximal tubule (14). This 12-transmembrane domain protein localizes to the apical membrane of proximal tubule epithelial cells and mediates urate reabsorption via exchange with Cl^−^ or organic anions. Antiuricosuric agents including lactate, pyrazinoate, and nicotinate act as exchange substrates for URAT1, thereby enhancing urate reabsorption (22). The apical transporter GLUT9 also plays a key role in UA reabsorption. Two major isoforms, hGLUT9a and hGLUT9b, arise from alternative splicing of the SLC2A9 gene (yielding transcripts SLC2A9a and SLC2A9b, respectively). GLUT9a exhibits broad tissue expression, whereas GLUT9b expression is primarily confined to key organs involved in urate transport and metabolism, such as the liver and kidneys (23). Notably, single nucleotide polymorphisms (SNPs) in the SLC2A9 gene are strongly associated with gout susceptibility, coronary artery disease, and myocardial infarction (24). ABCG2, an ATP-binding cassette (ABC) efflux transporter, functions as a high-capacity urate exporter mediating urate elimination at renal and extrarenal sites (25, 26). Genetic variation in human ABCG2 is a major contributor to HUA. Given that impaired renal urate excretion underlies HUA in most gout patients, deficient ABCG2-mediated renal urate secretion directly promotes HUA by reducing urate secretion and consequently increasing net renal urate reabsorption (27).

Critically, HUA resulting from transporter dysfunction leads to supersaturation and subsequent formation of MSU crystals, the essential danger signal that engages the NLRP3 inflammasome (2, 28). Under hyperuricemic stress, thioredoxin-interacting protein (TXNIP) is upregulated and serves as a key molecular bridge: it dissociates from the TRX complex in a ROS-dependent manner, directly binding to and activating NLRP3, while simultaneously modulating GLUT9 expression and urate reabsorption (29). Moreover, soluble uric acid upregulated expression of ABCG2 by activating the TLR4/NLRP3/caspase-1 inflammasome (30), suggesting a feedback loop between inflammatory activation and urate transporter regulation. This pathogenic connection is further supported by therapeutic studies demonstrating that interventions targeting both urate transporters and NLRP3 inflammasome activation can simultaneously lower serum urate and attenuate gouty inflammation (31). Together, these two core mechanisms—purine metabolic abnormalities and urate transporter dysfunction—establish the metabolic foundation for MSU crystal formation, the critical danger signal that subsequently engages the NLRP3 inflammasome.

### 2.2 Biophysical crystallization – from solute to danger signal

The transformation of UA from a dissolved solute into pathogenic crystals is governed by fundamental biophysical principles. Supersaturation occurs when serum UA concentration exceeds the thermodynamic solubility threshold (>6.8 mg/dL under physiological conditions), establishing a necessary condition for MSU crystal formation. This process is dynamically modulated by local microenvironmental factors, including temperature, pH, ionic strength, and the presence of nucleation agents (such as collagen fibres) (2, 32). Crucially, nucleation – the process of new microcrystal precipitation – is the rate-limiting step in MSU crystal generation. During nucleation, dispersed solute molecules first aggregate to form clusters, overcoming the solvent’s dispersive forces; these clusters subsequently coalesce to form crystal nuclei (13).

**Figure 1:**
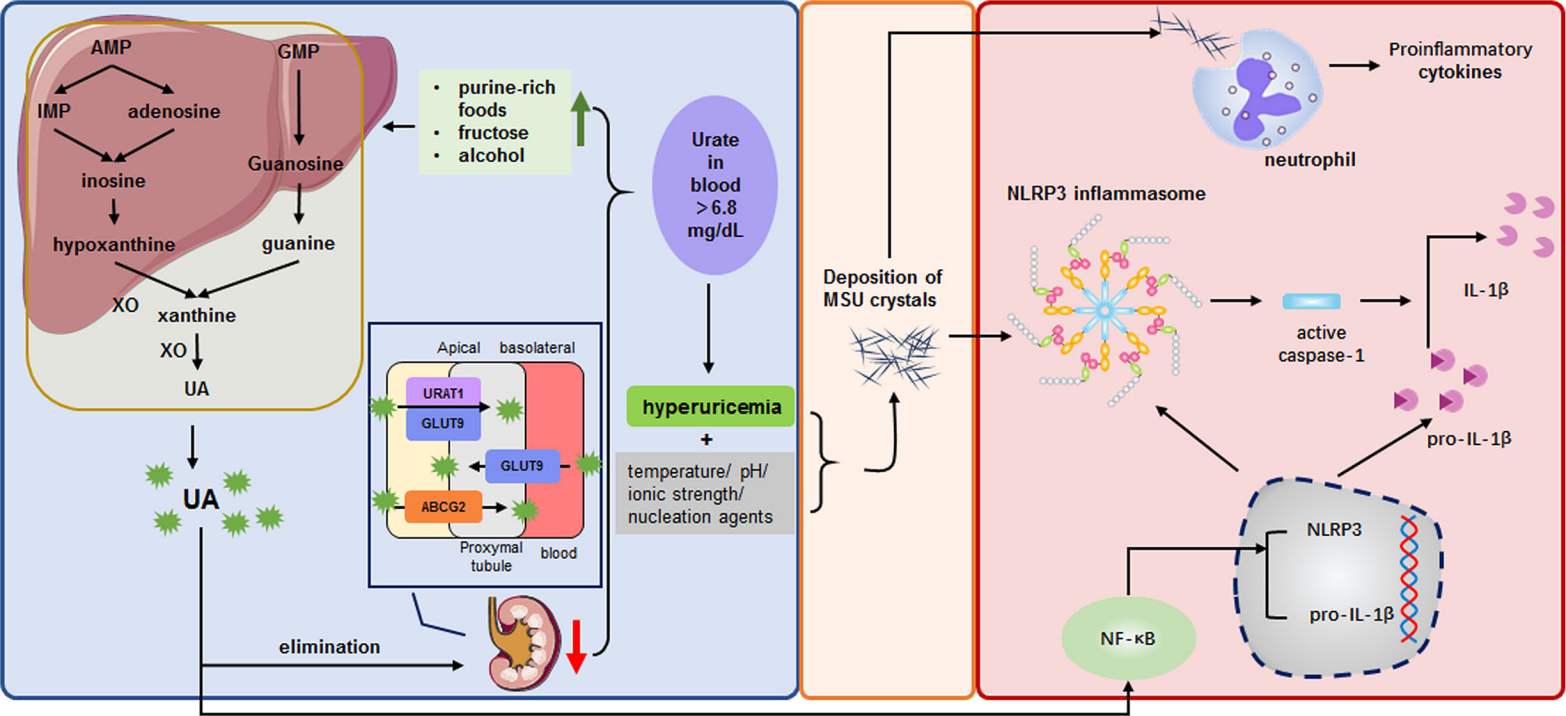
MSU crystals as a bridge linking metabolic dysregulation and innate immunity in gout. This schematic illustrates the pathogenic cycle of gout, in which MSU crystal deposition forms a critical bridge between systemic metabolic dysregulation and the activation of the innate immune response. Left Panel (Metabolic Dysregulation): HUA arises from uric acid overproduction and/or impaired renal excretion (mediated by dysfunction in urate transporters URAT1, GLUT9, and ABCG2). A sustained supersaturated state of uric acid (>6.8 mg/dL) leads to the nucleation and formation of MSU crystals. Center (Biophysical Crystallization): The formation of MSU crystals represents the pivotal transition from a soluble metabolic byproduct to a solid-phase danger signal. Right Panel (Innate Immune Activation): Deposited MSU crystals act as DAMPs, triggering two major pro-inflammatory pathways: (1) Macrophage NLRP3 Inflammasome Activation: Crystal phagocytosis induces lysosomal destabilization, potassium (K^+^) efflux, and NLRP3 inflammasome assembly, resulting in caspase-1-dependent maturation and release of pro-inflammatory cytokines. (2) Neutrophil Recruitment and Amplification: Opsonization by complement proteins and immunoglobulins promotes neutrophil chemotaxis, phagocytosis, and the release of NETosis, which amplifies the inflammatory response. These pathways culminate in an acute gout flare.

MSU crystal deposition is causally linked to the onset and progression of GA. These needle-shaped crystals deposit preferentially in avascular tissues (e.g., articular cartilage, tendons) and synovial fluid (32). Upon deposition, MSU crystals transform from inert structures into potent endogenous danger signals (DAMPs) (7). MSU crystal directly interact with macrophage membranes, triggering lysosomal rupture and potassium efflux, which further activates the NLRP3 inflammasome. This activation drives caspase-1-dependent maturation and release of IL-1β (7). Concurrently, opsonization by complement proteins (C3a, C5a) and IgG promotes neutrophil chemotaxis (33). Neutrophil phagocytosis of crystals further destabilizes phagolysosomes, amplifying NETosis and the release of proteolytic enzymes (e.g., myeloperoxidase), thereby establishing a self-perpetuating inflammatory cycle characteristic of acute gout (34). Critically, soluble urate primes the innate immune system by upregulating NLRP3 and pro-IL-1β expression in monocytes via NF-κB signaling, synergizing with crystallinity to amplify inflammation (35). Chronically, MSU deposits drive structural damage through: (1) mechanical erosion by tophi (36), (2) sustained macrophage-mediated inflammation and reactive oxygen species (ROS) release (37, 38), and (3) fibroblast-dependent synovitis and tissue remodeling (39). This established pathophysiology underscores the necessity of sustained urate-lowering therapy—targeting serum urate <6.8 mg/dL—to dissolve existing crystals, prevent *de novo* formation, and abrogate the cycle of sterile inflammation (**Figure 1**).

## 3 NLRP3 Inflammasome activation: a pivotal event in GA

GA represents a quint essential sterile inflammatory cascade driven by MSU crystal deposition, wherein the NLRP3 inflammasome functions as a key mechanistic hub. This hub integrates diverse danger signals originating from intra-articular MSU crystals and transduces them into a potent, IL-1β-centered cytokine storm. Its central role extends beyond mere activation; it orchestrates the initiation, amplification, and perpetuation of the inflammatory cascade that defines the acute gout flare phenotype. Consequently, elucidating the precise mechanisms governing NLRP3 inflammasome activation and its downstream sequelae is fundamental to understanding gout pathogenesis and developing targeted therapeutic interventions.

### 3.1 Molecular architecture and assembly of the NLRP3 inflammasome

The NLRP3 inflammasome is a cytosolic, multi-protein signaling complex comprising three core components (40): (1) The sensor protein NLRP3, characterized by a modular architecture featuring: an auto-repressive Leucine-Rich Repeat (LRR) domain that sterically occludes the central NACHT domain; the NACHT domain—a nucleotide responsible for oligomerization; and an N-terminal Pyrin domain (PYD) that mediates homotypic PYD-PYD interactions (41). (2) The adaptor protein ASC (Apoptosis-associated Speck-like protein containing a CARD), which possesses both a PYD domain (recruited to oligomerized NLRP3) and a Caspase Activation and Recruitment Domain (CARD), enabling its prion-like polymerization into filamentous “specks” upon activation (42). (3) The effector zymogen pro-caspase-1, which utilizes its N-terminal CARD domain for ASC-dependent recruitment (43, 44).

NLRP3 inflammasome assembly constitutes a highly orchestrated, two-step cascade process. The crucial initial step, termed priming (Signal 1), provides the necessary molecular groundwork. This inflammatory cascade is predominantly initiated when pattern recognition receptors (PRRs) recognize MSU crystals or co-released endogenous damage-associated molecular patterns (DAMPs). Among these PRRs, Toll-like receptors, particularly TLR2 and TLR4, play a central role in this initial recognition step. Engagement of these receptors activates downstream signaling cascades, chiefly the nuclear factor kappa B (NF-κB) pathway (45). NF-κB translocation to the nucleus induces the transcriptional upregulation of key components required for inflammasome function: the NLRP3 gene itself, the inactive precursor forms of the effector cytokines (pro-IL-1β), and potentially other inflammasome-associated proteins (46). Priming does not directly trigger inflammasome assembly but dramatically lowers the threshold for activation by subsequent stimuli and ensures the availability of substrates. This step effectively “primes” the cell, making it exquisitely sensitive to the danger posed by the crystals. The second and indispensable step is activation (signal 2). This process is directly triggered by diverse activating factors, such as environmental bacteria, viruses, and dust particles, as well as intracellular metabolites and substances released upon cell death within the body (47). These factors induce severe cellular stress via activated macrophages, generating multiple converging danger signals that ultimately trigger NLRP3 inflammasome assembly and activation. This activation leads to caspase-1 activation, which cleaves the precursors of IL-1β and IL-18 (46), thereby inducing the maturation and secretion of various inflammatory cytokines (48). This process also triggers pyroptosis, a pro-inflammatory form of programmed cell death (49). Additionally, a non-canonical NLRP3 inflammasome activation pathway exists. During Gram-negative bacterial infections, bacterial components can activate caspase-11 (caspase-4/5 in humans), which subsequently activates caspase-1, culminating in pyroptosis (50).

Despite diverse NLRP3 inflammasome agonists, the molecular mechanisms governing its activation remain incompletely elucidated. Three predominant hypotheses are recognized: potassium efflux, mitochondrial reactive oxygen species (ROS) production, and lysosomal rupture. Among these, potassium efflux serves as a critical upstream trigger, sufficient to activate NLRP3 (51). Canonical agonists (e.g., ATP, nigericin) induce plasma membrane K^+^ efflux and pro-IL-1β processing, with reduced intracellular K^+^ correlating with activation (52). Direct evidence demonstrates that a decrease in intracellular potassium concentration alone is sufficient to activate NLRP3. Moreover, experimental depletion of intracellular K^+^ by incubation in potassium-free medium can initiate inflammasome assembly independently of other stimuli, establishing K^+^ efflux as a universal and essential signal for NLRP3 inflammasome activation (53). ROS production represents another key mechanism. Mitochondria are organelles that make significant contributions to the production of ROS within cells, cellular stimulation induces mitochondrial damage, releasing ROS that activates TXNIP. Activated TXNIP binds NLRP3 to trigger inflammasome assembly (54), demonstrating the essential role of mitochondrial ROS in NLRP3 activation. Accumulating evidence underscores the essential role of mitochondrial ROS in NLRP3 activation—treatment with specific mitochondrial ROS scavengers (e.g., Mito-TEMPO) or mitochondrial-targeted antioxidants abolishes NLRP3 inflammasome activation in response to diverse stimuli (55), confirming the indispensable role of mitochondria-derived ROS in this process. Lysosomal rupture constitutes the third major pathway. Large crystalline activators undergo endocytosis but exceed the degradative capacity of phagolysosomes, resulting in lysosomal rupture. Pharmacological inhibition of cathepsin B with CA-074-Me or genetic deficiency of cathepsin B significantly impairs MSU and cholesterol crystal-induced IL-1β secretion (56). Furthermore, direct lysosomal disruption with the dipeptide L-leucyl-L-leucine methyl ester (Leu-Leu-OMe) is sufficient to trigger NLRP3 inflammasome activation independently of crystalline stimuli (57), establishing lysosomal destabilization and subsequent cathepsin release as a direct activation mechanism.

Beyond the three classical activation pathways, recent studies have uncovered several novel regulatory mechanisms of NLRP3 inflammasome activation. NEK7 has been identified as an essential licensing factor that directly binds to the LRR domain of NLRP3 following potassium efflux, thereby bridging ionic signals to inflammasome oligomerization and activation (58). Furthermore, NLRP3 was shown to be recruited to the dispersed trans-Golgi network (dTGN) through ionic bonding between its conserved polybasic region and negatively charged phosphatidylinositol-4-phosphate (PtdIns4P), where the dTGN serves as a scaffold to promote NLRP3 aggregation and ASC polymerization (59). Recent advances have substantially expanded this view by unveiling critical roles for diverse post-translational modifications (PTMs) and dynamic subcellular localization (60). Beyond well-studied phosphorylation and ubiquitination, novel PTMs including SUMOylation (61), palmitoylation (62), acetylation (63), ISGylation (64) and alkylation (65) have been identified as crucial checkpoints during both the priming (Signal 1) and activation (Signal 2) phases. Mechanistically, these modifications regulate NLRP3 stability, protein-protein interactions, and membrane association—for instance, site-specific palmitoylation by ZDHHC enzymes directs NLRP3 trafficking to distinct membrane compartments, while phosphorylation events govern its interaction with NEK7. Concurrently, the subcellular localization of NLRP3 is crucial for its assembly process. Its dynamic distribution within structures including the endoplasmic reticulum (66), mitochondria (67), endosomes (68), and microtubule organizing centers (69) enables precise spatial regulation of NLRP3 oligomerization and downstream signaling. These advances collectively expand our understanding of the molecular checkpoints governing NLRP3 activation and offer novel therapeutic opportunities for gout and other inflammatory diseases.

### 3.2 NLRP3 inflammasome in GA pathogenesis

During GA pathogenesis, MSU crystal deposition triggers acute flares characterized by rapid-onset painful joint swelling with localized erythema and heat (70). Histologically, these flares feature pronounced neutrophilic infiltration in synovial tissue and fluid (71). This inflammatory cascade initiates when uric acid—released from damaged cells as an endogenous danger signal—primes T cell-mediated immunity (72). Mechanistically, phagocytosed MSU crystals activate the NLRP3 inflammasome in resident macrophages, driving caspase-1-dependent proteolytic maturation of pro-IL-1β (7, 73). Notably, MSU crystals alone are insufficient to induce robust IL-1β expression and require synergistic secondary signals (e.g., LPS) to prime inflammation (74), and Toll-like receptors (TLRs) contribute critically to this priming phase. Research found that genetic ablation of TLR2 or TLR4 suppresses MSU-induced IL-1β production and neutrophil influx in murine models (75). However, MyD88-dependent IL-1R signaling maintains acute inflammation even when TLR2/4 pathways are disrupted (76).

**Figure 2:**
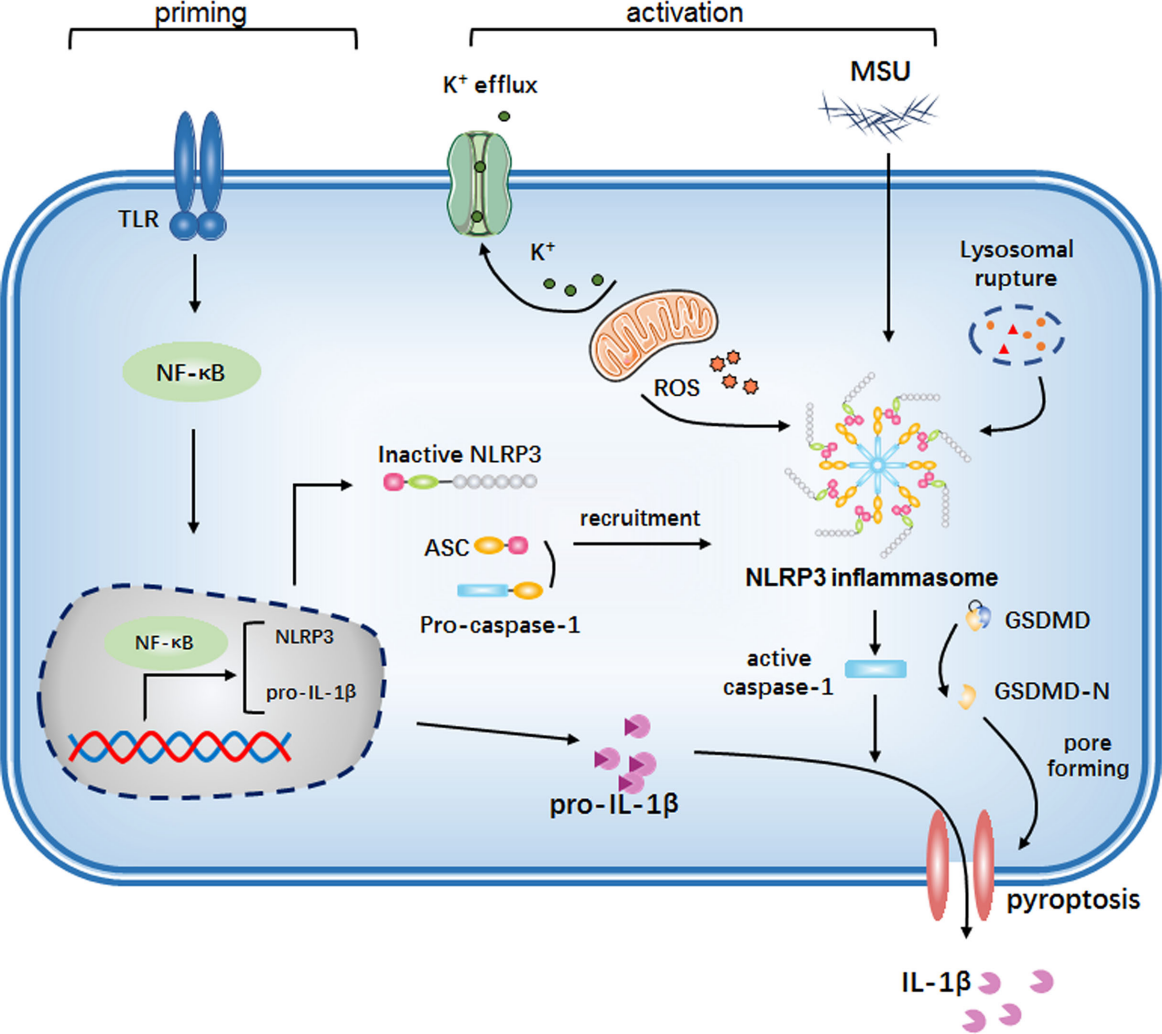
The NLRP3 inflammasome is activated by MSU crystals. First, cells detect endogenous or exogenous molecules that activate Toll-like receptors (TLRs) or other pattern recognition receptors, leading to the activation of the intracellular nuclear factor-κB (NF-κB) signaling pathway. This activation promotes the transcription and expression of both NLRP3 and pro-IL-1β. Subsequently, monosodium urate (MSU) crystals are recognized by the cell as an activation signal, triggering the assembly of the NLRP3 inflammasome. This in turn activates caspase-1, which cleaves the IL-1β precursor into its mature form. This process induces the maturation and secretion of various inflammatory cytokines, accompanied by the induction of pyroptosis.

Central to gout pathogenesis is the activation of mononuclear phagocytes, which drive interleukin-1β (IL-1β) production—emerges as the principal inflammatory mediator driving the acute gout flare (77). It initiates a potent, self-amplifying inflammatory cascade: upon its generation during NLRP3 inflammasome activation, IL-1β triggers vasodilation of the endothelium at inflammatory sites and recruits IL-1R-expressing cells—such as monocytes, resident macrophages, and neutrophils—to arthritic joints through the action of multiple chemokines including CXCL8/IL-8 and MCP-1 (78). The release of IL-1β in response to MSU crystals is fundamentally dependent on NLRP3 inflammasome activation, as demonstrated *in vitro* (79). Furthermore, *in vivo* evidence solidifies this link: NLRP3 deficiency significantly attenuates MSU-induced inflammation and gout symptoms in murine models (7), definitively establishing the NLRP3 inflammasome’s central role in GA. The indispensable nature of the IL-1 pathway is further underscored by studies showing significant suppression of MSU-induced inflammation in IL-1 receptor-deficient mice and in wild-type mice treated with IL-1 inhibitors (76). Collectively, these findings validate the NLRP3-caspase-1-IL-1β axis as a core pathogenic mechanism and a prime therapeutic target for gouty arthritis (**Figure 2**).

Thus, the NLRP3 inflammasome functions as the central signaling hub that integrates the “danger” signature of MSU crystals and transduces it into IL-1β-driven inflammation, directly mediating the cardinal manifestations of acute gout—rapid-onset pain, swelling, erythema, and warmth. This pivotal role positions NLRP3 as a prime therapeutic target. Current therapeutic strategies, including IL-1β inhibitors (e.g., anakinra, canakinumab) and NLRP3 inhibitors (e.g., MCC950, dapansutrile), target this core pathogenic axis.

### 3.3 Genetic variants of NLRP3 in gout susceptibility and progression

Increasing evidence suggests that genetic predisposition plays a significant role in the onset and progression of gout. Among key genetic factors, polymorphisms in the gene encoding the core inflammasome sensor protein NLRP3 have been extensively studied. These genetic variants are closely associated with susceptibility to gout, disease severity, and clinical manifestations.

Studies across different populations have identified specific NLRP3 single nucleotide polymorphisms (SNPs) linked to GA or HUA risk. To clearly present the findings from different loci, the key results are summarized in **Table 1**.

**Table 1:** Association studies of NLRP3 gene polymorphisms with gout/HUA.

NLRP3 polymorphic locus	Associated disease	Functional effect	Population	Reference
rs3806268	GA	GG genotype increases risk of primary gout; may contribute to pathophysiology via immune response regulation	Chinese population	(80, 81)
rs3738448	GA/HUA	T allele may enhance NLRP3 mRNA stability; potentially involved in gout pathogenesis through immune regulation	Chinese population	(80)
rs7525979	HUA	TT genotype associated with increased HUA risk in subgroups with hypertension and no alcohol consumption, compared to CC genotype	Chinese population	(80)
rs10754558	GA	GG genotype is a significant risk factor; may influence immune and inflammatory responses by modulating NLRP3 inflammasome component expression	Chinese Han population	(82)
rs12239046, rs7512998, rs12137901, rs12565738	GA	No statistically significant relevance	Chinese population	(81)
rs4612666	GA	No statistically significant relevance	Chinese/Chinese Han population	(81, 82)
rs1539019	GA	No statistically significant relevance	Chinese Han population	(82)
rs7512998, rs12137901	GA	No statistically significant relevance	Chinese population	(83)

In summary, specific NLRP3 polymorphisms demonstrate a significant association with gout susceptibility, supporting their potential as both candidate genetic markers and susceptibility factors for the disease. Critically, functional evidence links these variants to enhanced inflammasome activity and IL-1β signaling, thereby underscoring the NLRP3 inflammasome’s indispensable role in gout pathogenesis.

## 4 Metabolic danger signals as the “primer” and “amplifier” of the NLRP3 inflammasome

The pathogenesis of GA represents a paradigm of sterile inflammation where the initial urate crystal deposition intersects profoundly with systemic and local metabolic dysregulation. While MSU crystals are the indispensable trigger for NLRP3 inflammasome activation and IL-1β release within the joint, the susceptibility, intensity, and persistence of the inflammatory response are critically modulated by the underlying metabolic environment. This section delves into the intricate molecular crosstalk between key metabolic pathways and the NLRP3 inflammasome mechanisms, elucidating how metabolic signals amplify, prime, or directly engage the inflammatory cascade central to gout flares and chronicity. A summary of the mechanisms by which these metabolic danger signals regulate the NLRP3 inflammasome is provided in **Table 2**.

**Table 2:** Mechanisms of key metabolic danger signals in regulating the NLRP3 inflammasome in gouty Arthritis.

Category	Metabolic danger signal	Primary source	Mechanism of action on NLRP3	Effect	References
Soluble Uric Acid (sUA)	Soluble Uric Acid (sUA)	HUA; Cellular catabolism	Priming: Acts as a DAMP; activating NF-κB pathway; transcriptionally upregulating NLRP3 and pro-IL-1β. Activation: Induces mitochondrial ROS production and facilitates K^+^ efflux; triggering NLRP3 inflammasome assembly.	Lowers NLRP3 activation threshold; directly promotes IL-1β maturation and secretion; suppresses IL-1Ra; exacerbating inflammation; increases the risk of acute gout flares and amplifies flare intensity.	(46, 84, 85)
Free Fatty Acids & Lipotoxicity	Saturated (SFA; e.g.; Palmitate; Stearate)	High-fat diet; Obesity; Alcohol consumption; Fasting; Metabolic Syndrome	Priming & Amplification: Stearate engages TLR2/MyD88 signaling driving sustained NF-κB activation; upregulating NLRP3 and pro-IL-1β. Synergistic Activation: Co-stimulation with MSU crystals synergistically promotes ASC/caspase-1-dependent IL-1β release. Direct Activation: Palmitate can directly activate the NLRP3 inflammasome.	Significantly enhances MSU crystal-induced inflammation; directly causes IL-1β and IL-18 production; potentiates acute gouty arthritis and contributes to flare persistence.	(45, 86, 91, 93)
	Unsaturated Fatty Acids (e.g.; Oleate; Linoleate)	Plant oils; Fish	Inhibition: Does not affect SFA-induced transcriptional priming but specifically inhibits the NLRP3 inflammasome assembly/activation step. Mechanisms may involve GPR40/120 receptor engagement leading to β-arrestin-2 binding to NLRP3 and/or mitigation of ER stress.	Effectively suppresses IL-1β release triggered by SFAs; MSU crystals; and other NLRP3 agonists; exerting an anti-inflammatory effect; may attenuate acute gout flare severity.	(94, 95)
Cholesterol& Oxidized LDL	Cholesterol	High-cholesterol diet; Dyslipidemia	CCs lead to lysosomal disruption; cathepsin B release; and direct NLRP3 activation. Intracellular Cholesterol Homeostasis: The SCAP-SREBP2 translocation process promotes NLRP3 activation.	Leads to caspase-1 activation and IL-1β/IL-18 maturation; driving neutrophil recruitment and chronic joint damage; exacerbates acute gout flare frequency and promotes gouty arthropathy progression.	(100, 101, 104)
	Oxidized LDL (oxLDL)	High-cholesterol diet; Dyslipidemia	Priming: Engages CD36/TLR4; activating NF-κB to upregulate NLRP3 and pro-IL-1β. Activation: Triggers assembly via impaired cholesterol efflux (via ABCA1); lysosomal rupture; ER stress; mitochondrial ROS; and K^+^ efflux.	Promotes foam cell formation; contributes to chronic synovial inflammation and recurrent gout flares.	(108)
Hyperglycemia & AGEs	Hyperglycemia	Diabetes; Insulin resistance	Mediates NLRP3 activation via ATP-P2X signaling. Upregulates NLRP3 and pro-caspase-1 expression. MSU crystals upregulate GLUT1 and glycolysis; promotes NLRP3-dependent IL-1β production.	Promotes IL-1β secretion; Elevates circulating uric acid and fatty acids; collectively exacerbating gouty inflammation.	(120, 121, 123)
	Advanced Glycation End Products (AGEs)	Diabetes; Persistent hyperglycemia	Binding to RAGE activates NF-κB signaling; providing transcriptional priming for NLRP3 activation.	Establishes a pro-inflammatory transcriptional foundation; acting synergistically with hyperglycemia; primes the joint environment for heightened inflammatory responses during MSU crystal exposure.	(125)

This table summarizes how key metabolic danger signals regulate NLRP3 inflammasome activity via distinct molecular mechanisms within the context of GA. ABCA1, ATP-binding cassette A1; AGE, advanced glycation end product; DAMP, damage-associated molecular pattern; oxLDL, oxidized low-density lipoprotein; RAGE, receptor for AGEs; ROS, reactive oxygen species; TLR, Toll-like receptor.

### 4.1 Soluble uric acid

HUA constitutes the fundamental prerequisite for GA, yet its pathological role extends far beyond serving as a mere precursor for MSU crystal formation. Elevated soluble uric acid (sUA) acts as a potent DAMP that actively engages the innate immune system. Specifically, sUA is recognized by TLRs on immune cells such as monocytes and macrophages (84). This binding triggers downstream signaling cascades that activate NF-κB, resulting in transcriptional upregulation of key inflammasome components, including *NLRP3* and *pro-IL-1β* (46), thereby effectively “priming” the inflammasome and lowering its activation threshold. It is noteworthy that MSU crystals and soluble uric acid exert opposing effects on IL-1 receptor antagonist (IL-1Ra): while MSU crystals—either alone or in concert with other ligands—enhance IL-1Ra secretion, sUA significantly suppresses it (84).

Furthermore, emerging evidence indicates that under specific pathological conditions, sUA can directly orchestrate NLRP3 inflammasome activation in a crystal-independent manner. Proposed mechanisms include induction of mitochondrial reactive oxygen species (ROS) and facilitation of potassium ion (K^+^) efflux (85). These events serve as critical secondary signals that promote ASC oligomerization, caspase-1 activation, and subsequent cleavage and secretion of mature IL-1β. This paradigm underscores that a persistent, low-grade pro-inflammatory state—driven by sUA—predates and may even promote subsequent MSU crystallization and the acute inflammatory outbursts characteristic of gout flares.

### 4.2 Free fatty acids and lipotoxicity

Dyslipidemia, a central feature of metabolic syndrome, contributes significantly to NLRP3 activation. Elevated free fatty acids (FFAs)—a hallmark of obesity and metabolic syndrome—serve as potent metabolic danger signals that critically prime and amplify NLRP3 inflammasome activation (86). Clinically relevant triggers of gout attacks, including abundant meal ingestion, alcohol consumption, and fasting, all acutely elevate circulating FFA concentrations (87, 88). This association is further supported by observations of increased FFA levels in gout patients during acute joint inflammation (89).

FFAs exert robust pro-inflammatory effects by directly engaging TLRs and inducing proinflammatory cytokine production (90). Specifically, stearate (C18:0) exerts pro-inflammatory effects by directly engaging TLR2 (91). This interaction activates the TLR2/MyD88 signaling pathway, which drives the priming step of NLRP3 inflammasome activation (45, 92). When FFAs and MSU crystals co-stimulate cells, synergistic ASC/caspase-1-dependent IL-1β release occurs, mechanistically linking metabolic changes preceding gout attacks to IL-1β-driven joint inflammation. Complementing this, palmitate (C16:0) directly activates the NLRP3 inflammasome, inducing IL-1β and IL-18 production in LPS-primed bone marrow-derived macrophages (93).

In stark contrast, unsaturated fatty acids (UFAs) such as oleic acid (C18:1) and linoleic acid (C18:2) not only fail to induce IL-1β secretion but actively suppress NLRP3 inflammasome activation. These UFAs effectively inhibit IL-1β release triggered by SFAs and, albeit less potently, by diverse NLRP3 activators including nigericin, alum, and MSU crystals (94). Crucially, UFAs do not impair the SFA-induced transcriptional upregulation of NLRP3 or pro-IL-1β, indicating that their inhibitory effect specifically targets the inflammasome assembly/activation step rather than upstream priming signaling. This suppression is proposed to involve mechanisms such as engagement of GPR40/120 receptors initiating β-arrestin-2 binding to NLRP3 and/or mitigation of endoplasmic reticulum stress (94–96). This opposing action highlights a critical regulatory axis where the balance of saturated versus unsaturated FFAs significantly modulates NLRP3 inflammasome activity and IL-1β-driven inflammation in GA.

### 4.3 Cholesterol and oxidized LDL

Accumulating evidence underscores the significant contribution of dysregulated lipid metabolism, particularly hypercholesterolemia, to the pathogenesis and severity of GA (97). While HUA and MSU crystal deposition define GA, its clinical manifestations—especially acute flare intensity and frequency—are increasingly linked to metabolic comorbidities such as dyslipidemia (98). Elevated serum cholesterol commonly coexists with HUA, suggesting intertwined metabolic derangements that extend beyond cardiovascular risk.

Critically, cholesterol acts not merely as a structural lipid but as a pathogenic mediator in GA. Similar to MSU crystals, cholesterol crystals (CCs) can also act as powerful endogenous danger signals to activate NLRP3 inflammasome (72, 99). Upon macrophage phagocytosis, CCs disrupt lysosomal integrity, triggering cathepsin B release and subsequent NLRP3 inflammasome assembly (100, 101), cholesterol accumulation in DCs also accelerate autoimmunity via NLRP3 (102, 103). Guo et al. discovered that intracellular cholesterol homeostasis promotes NLRP3 inflammasome activation via the SCAP-SREBP2 translocation process from the endoplasmic reticulum to the Golgi apparatus (104). Moreover, recent evidence suggests that cholesterol transport-induced NLRP3 activation is mediated by the CaMKII/JNK pathway, which promotes NLRP3 deubiquitination (105), while complement activation by CCs trigger mitochondrial dysfunction and ROS production (106). This cascade culminates in caspase-1 activation and proteolytic maturation of IL-1β/IL-18—core cytokines driving neutrophil recruitment, vascular leakage, and chronic joint damage in gout (100, 101).

Oxidized LDL (oxLDL) propagates inflammation through dual mechanisms: transcriptional priming and inflammasome activation. Primarily, oxLDL promotes NLRP3 inflammasome priming in macrophages through engagement of scavenger receptors (CD36/TLR4) (100, 107). Furthermore, oxLDL triggers NLRP3 inflammasome assembly via multiple downstream effects: impairing cholesterol efflux through ATP binding cassette A1 (ABCA1), inducing lysosomal disruption (increasing cathepsin B activity), promoting ER stress, causing mitochondrial damage and elevated ROS, and enhancing potassium efflux (108–110). Conversely, pharmacological inhibition of the NLRP3 inflammasome reduces the formation of cholesterol-laden foamy macrophages (111, 112).

The crosstalk between cholesterol metabolism and NLRP3 inflammasome activation represents a critical pathogenic axis in gout, offering a mechanistic basis for the aggravation of joint inflammation in patients with comorbid dyslipidemia (100). Future studies could aim to clarify the regulation of these interactions within the joint microenvironment, and explore whether modulating cholesterol homeostasis or NLRP3 activity can alter the natural history of gout. Combining lipid-modifying agents with anti-inflammatory treatments might thus represent a promising translational approach for severe or refractory gout, particularly in metabolically compromised patients.

### 4.4 Hyperglycemia and advanced glycation end products

Hyperglycemia and gout are frequently comorbid conditions within metabolic syndrome and exhibit a well-established bidirectional relationship. Large-scale epidemiological studies consistently support this association. For instance, data from the US National Health and Nutrition Examination Survey revealed a diabetes prevalence of 25.7% among individuals with gout compared to 7.8% in those without (113). Similarly, a UK cohort study reported that patients with type 2 diabetes mellitus (T2DM) have a 48% increased risk of developing gout compared to non-diabetic controls (114). Although this elevated risk has often been attributed to common comorbidities such as obesity, renal impairment, and hypertension, growing evidence implicates shared pathophysiological mechanisms (114). Chronic inflammation, insulin resistance, and hyperinsulinemia are hallmarks of both hyperuricemic gout and T2DM, suggesting common underlying pathways (115, 116). Central to this interplay is the aberrant activation of the NLRP3 inflammasome (117).

During acute infection or tissue injury, metabolic reprogramming analogous to the Warburg effect occurs to support phagocytic and inflammatory functions (118). Enhanced glucose uptake and glycolysis are metabolic features of pro-inflammatory immune activation. High glucose promotes IL-1β production via an NLRP3-dependent mechanism (119). ATP-P2X signaling mediate high glucose-induced NLRP3 activation and IL-1 family cytokine secretion (120). Furthermore, high glucose upregulates NLRP3 and procaspase-1 expression in mesangial cells *in vitro* and *in vivo*, reinforcing inflammasome activation (121). Sustained hyperglycemia also elevates circulating uric acid and fatty acids (122), contributing to GA. MSU crystals amplify glucose uptake in macrophages by upregulating GLUT1 and enhancing glycolytic activity. This metabolic reprogramming promotes NLRP3-dependent IL-1β production (123) (124). Accordingly, inhibition of GLUT1 or glycolysis attenuates MSU-induced inflammation. Persistent hyperglycemia also accelerates the formation of advanced glycation end products (AGEs). AGE engagement with RAGE activates NF-κB signaling and further providing essential transcriptional priming for NLRP3 inflammasome activation (125).

In summary, hyperglycemia is not a mere comorbidity but actively exacerbates gouty inflammation by priming and activating the NLRP3 inflammasome through multiple mechanisms. Consequently, the comprehensive management of gout should extend beyond conventional urate-lowering therapy to include targeted glycemic control as an integral component of treatment strategies.

## 5 Targeting NLRP3 inflammasome by regulating metabolic signaling pathways: a novel therapeutic strategy for gouty arthritis

Although current first-line treatments for GA primarily include urate-lowering agents (e.g., allopurinol and febuxostat) and anti-inflammatory drugs (such as colchicine, nonsteroidal anti-inflammatory drugs, and IL-1 inhibitors)—which are effective at reducing serum UA levels and managing acute inflammation—their therapeutic effects are largely limited to certain stages of the disease. These treatments are also constrained by adverse drug reactions, suboptimal treatment adherence, and a high rate of disease recurrence. Recent advances in GA pathogenesis research have elucidated the critical role of MSU crystal-induced activation of the NLRP3 inflammasome and subsequent IL-1β-mediated inflammatory responses, motivating a shift toward therapeutics that specifically inhibit this signaling cascade. Furthermore, mounting evidence highlighting the interplay between metabolic dysregulation and innate immune activation has fundamentally reshaped our understanding of GA pathogenesis, prompting the development of novel treatment strategies focused on immunometabolic modulation. **Table 3** summarizes small-molecule compounds that exert anti-GA effects by regulating metabolic signaling pathways and the NLRP3 inflammasome.

**Table 3:** Small-molecule compounds targeting the NLRP3 inflammasome and related pathways for the intervention of gouty arthritis.

	Name	Target	Anti- inflammatory mechanism	Reference
Direct NLRP3 Inhibitors	OLT1177	NLRP3 NACHT domain	Inhibits the NLRP3 inflammasome by targeting NACHT domain, and further reduces atpase activity, prevents NLRP3 self-oligomerization, ASC speck formation, and pro-caspase-1 recruitment	(126)
	Tranilast	NLRP3 NACHT domain	Directly binds to the NACHT domain, disrupting NLRP3–ASC and NLRP3–NLRP3 interactions and inhibiting oligomerization	(131)
	Oridonin	NLRP3 NACHT domain	Covalent binding to cysteine 279 within the NACHT domain, disrupting the NLRP3–NEK7 interaction	(132)
IL-1	Firsekibart	IL-1β	A humanized monoclonal antibody. Specifically neutralizes il-1β by blocking its interaction with the il-1 receptor, inhibiting downstream inflammatory signaling.	(134, 135)
	Canakinumab	IL-1β	A fully human anti–IL-1β monoclonal antibody, selectively binds to IL-1β and prevents its interaction with the receptor	(138, 139)
	Anakinra	IL-1 receptor	A recombinant IL-1 receptor antagonist. Competitively inhibits the binding of IL-1α and IL-1β to the IL-1 receptor, attenuating IL-1-mediated signaling	(141, 142)
	Rilonacept	IL-1α/IL-1β	A soluble receptor-Fc fusion protein. Inhibiting both IL-1α and IL-1β by blocking their binding to cell surface receptors	(145, 146)
Uric Acid Homeostasis	Febuxostat	XOR	Inhibits the activity of XOR oxidase and dehydrogenase; suppresses NLRP3-driven IL-1β release and macrophage death by restoring intracellular ATP levels and mitochondrial function.	(152, 153)
	Allopurinol	XOR	A hypoxanthine analog, competitively inhibits XOR, thereby reducing uric acid production.	(150)
Fatty Acid Synthesis	C75	FASN	Reduce ROS levels and suppress pyroptosis via activation of the Nrf2/HO-1 pathway. Downregulates the expression of NLRP3, Caspase-1, IL-1β, and IL-18	(158)
	Trimetazidine	FAO	Inhibiting FAO with trimetazidine reduces NLRP3 inflammasome activation	(161)
	ω-3 PUFAs	lipid-lowering effects	Downregulating the expression of inflammasome-related genes in adipocytes and macrophages, effectively suppresses NLRP3 inflammasome activation	(95, 163, 164)
Lipid Metabolism	Statins	HMG-CoA Reductase	Inhibit oxldl- or TNF-α-induced NLRP3 inflammasome activation	(166)
	Probucol	Oxidative Stress	Uppresses NLRP3 inflammasome activation, NF-κB signaling, and downstream proinflammatory cytokines including IL-1β, TNF-α, and IL-6	(169)
	Ezetimibe	NPC1L1	Attenuate NLRP3–IL-1β pathway activation in an autophagy-dependent mechanism	(171)
	Parthenolide	Caspase-1	Directly targets caspase-1 to suppress inflammasome activity	(172)
	Glycyrrhizin	NLRP3	Broad anti-inflammatory effects by inhibiting NLRP3 inflammasome activation	(173)
	Curcumin	NF-κB/Mitochondria/NLRP3	Suppressing IκBα degradation, NF-κB activation, mitochondrial dysfunction, and NLRP3 inflammasome activity	(174)
Glycolysis	Metformin	AMPK	Inhibits NLRP3 inflammasome activation via the AMPK/mtor signaling pathway	(179)
	2-DG	Glycolysis	Attenuates NLRP3 activation, potentially through alleviating oxidative stress	(180)
	Shikonin	PKM2	Suppresses the PKM2–EIF2AK2 pathway, thereby attenuating NLRP3 and AIM2 inflammasome activation and reducing the secretion of IL-1β, IL-18, and HMGB1	(182)
	GSK2837808A	LDHA	Suppressed NLRP3 activation	(183)
	Chaetocin	histone methyltransferase	Inhibits the expression of HIF-1α and hexokinase 2, and subsequently suppress NLRP3 inflammasome activation	(184)

### 5.1 Targeting the NLRP3-IL-1β axis

Based on the elucidation of interactions between metabolic signaling pathways and the NLRP3 inflammasome, multiple potential therapeutic targets have emerged for the treatment of gouty arthritis. Among these, the NLRP3 inflammasome itself represents a highly promising target.

#### 5.1.1 Direct NLRP3 inhibitors

OLT1177 (Dapansutrile) is an orally bioavailable β-sulfonyl nitrile compound that selectively inhibits the NLRP3 inflammasome by targeting its NACHT domain. This interaction reduces ATPase activity and prevents NLRP3 self-oligomerization, ASC speck formation, and pro-caspase-1 recruitment, ultimately suppressing caspase-1 activation and the release of IL-1β and IL-18 (126). In a randomized, double-blind, placebo-controlled phase II trial involving patients with acute gout flares, treatment with OLT1177 resulted in 82% of patients achieving target joint pain scores ≤2 by Day 3, with complete flare resolution by Day 7 (127). A subsequent dose-finding trial identified 200 mg twice daily as the optimal regimen, demonstrating 52.4–68.4% pain reduction by Day 3 and 68.9–84.2% by Day 7 across dose groups, with 88% of patients achieving complete flare resolution by Day 8 (128). Both studies reported favorable safety profiles with no drug-related serious adverse events. When compared to traditional anti-inflammatory agents such as NSAIDs and colchicine—which are often contraindicated in patients with chronic kidney disease or cardiovascular comorbidities—OLT1177 offers the advantage of targeted NLRP3 inhibition without broad immunosuppression or organ toxicity (129). As the most advanced direct NLRP3 inhibitor in clinical development for gout, its oral bioavailability and specific mechanism of action could represent a paradigm shift in the management of acute flares, circumventing the broad immunosuppression associated with conventional anti-cytokine therapies.

Tranilast, originally an anti-allergic agent, has been repurposed as a dual-mechanism drug for gout management. It directly inhibits the NLRP3 inflammasome by binding to its NACHT domain, thereby blocking NLRP3 oligomerization and subsequent IL-1β release (108). Concurrently, tranilast exerts potent uricosuric effects by inhibiting major renal urate reabsorption transporters, including URAT1, GLUT9, OAT4, and OAT10, providing a molecular basis for its serum uric acid-lowering activity (130). Preclinical studies demonstrate its ability to reduce MSU crystal-induced IL-1β secretion in macrophages and mitigate gout-related inflammation in animal models (131). Multiple phase II trials have evaluated tranilast in gout and HUA. The TAnGO study (NCT01109121)—a multicenter, randomized, double-blind, phase II trial enrolling 112 patients with moderate to severe gout and HUA—assessed tranilast 300 mg combined with allopurinol (400 or 600 mg) versus allopurinol alone over four weeks. The primary outcome was percent change from baseline in serum uric acid at week 4, with secondary outcomes including the proportion of patients achieving sUA <6.0 mg/dL. Additional phase II studies have examined tranilast alone or combined with xanthine oxidase inhibitors (allopurinol, febuxostat) in hyperuricemic patients (NCT01052987, NCT00995618). These completed trials support tranilast’s potential as a dual-action therapeutic combining anti-inflammatory and urate-lowering effects, though further studies are needed to establish its efficacy specifically in gout populations.

Oridonin, a bioactive compound derived from Rabdosia rubescens, also inhibits NLRP3 inflammasome activation via covalent binding to cysteine 279 within the NACHT domain, thereby disrupting the NLRP3–NEK7 interaction. It has shown both preventive and therapeutic effects in murine models of gouty arthritis (132). Similarly targeting the NACHT domain but through a distinct mechanism, CY-09 is a selective small-molecule inhibitor that binds to the ATP-binding motif, inhibiting NLRP3 ATPase activity and preventing inflammasome oligomerization and subsequent IL-1β release. Although no clinical trials have been conducted to date, robust preclinical evidence supports its therapeutic potential for gout: ex vivo studies demonstrated that CY-09 significantly reduced IL-1β secretion in synovial fluid cells isolated from gout patients, while *in vivo* treatment effectively suppressed MSU-induced IL-1β production and neutrophil infiltration in murine models of acute gouty arthritis. Collectively, these preclinical compounds validate NLRP3 as a druggable target and provide promising leads for future therapeutic development in gout (133).

#### 5.1.2 Targeting IL-1

Firsekibart is a humanized monoclonal antibody that specifically neutralizes IL-1β by blocking its interaction with the IL-1 receptor, thereby inhibiting downstream inflammatory signaling. Unlike conventional symptomatic treatments, firsekibart demonstrates efficacy in both acute and intercritical phases of gouty arthritis. It not only rapidly alleviates acute symptoms but also significantly reduces long-term recurrence risk. Clinical studies report that a single dose provides analgesia comparable to corticosteroids within 6–72 hours and reduces the six-month recurrence risk by nearly 90%, with no serious drug-related adverse events (134, 135). Approved in June 2025, firsekibart offers a new option for acute gouty arthritis in patients with contraindications or intolerance to NSAIDs, colchicine, or steroids.

Canakinumab is a fully human monoclonal antibody that targets IL-1β with high affinity and selectivity, preventing its interaction with the IL-1 receptor and thereby providing potent and long-lasting neutralization of IL-1β-mediated inflammation (136). Large-scale randomized controlled trials have established its superiority compared to triamcinolone in pain relief, reduction of inflammation, and prevention of gout flares, particularly among patients with contraindications or inadequate response to NSAIDs and/or colchicine (137, 138). For instance, Schlesinger et al. (139) demonstrated that a single 150 mg subcutaneous dose of canakinumab was more effective than a single 40 mg intramuscular injection of triamcinolone acetonide in relieving pain at the 72-hour mark.

Anakinra is a recombinant IL-1 receptor antagonist that targets the IL-1 receptor by competitively inhibiting the binding of both IL-1α and IL-1β, thereby attenuating IL-1-mediated inflammatory signaling (140). Although initially approved for rheumatoid arthritis, it is now widely used off-label for the treatment of gout. Clinical trials and large case series have established its efficacy and safety in managing acute gout flares, demonstrating non-inferiority to conventional therapies (141). Across multiple outcome measures, anakinra consistently exhibited numerically superior efficacy compared to standard care. Notably, anakinra provides a valuable therapeutic alternative for difficult-to-treat gout patients, including those with comorbidities such as diabetes, renal impairment, or cardiovascular disease. This is supported by a retrospective case series involving 31 patients with renal transplantation or advanced chronic kidney disease, in which gout flare symptoms resolved following anakinra treatment (142). Furthermore, a comparative study showed that while anakinra was not superior to triamcinolone in achieving the primary endpoint, it offered comparable pain reduction and was favored in most secondary endpoints (143). Despite its short half-life, which necessitates daily subcutaneous administration, the targeted mechanism of anakinra makes it a clinically relevant biological option for gout management.

Rilonacept is a soluble receptor-Fc fusion protein that inhibits both IL-1α and IL-1β by blocking their binding to cell surface receptors (144). Clinical studies have demonstrated that rilonacept significantly reduces median symptom- and severity-adjusted joint scores and markedly lowers high-sensitivity C-reactive protein levels compared to placebo (145). Furthermore, rilonacept has been evaluated for the prevention of acute flares following the initiation of urate-lowering therapy (ULT), showing a significant reduction in flare incidence relative to placebo. Its efficacy in reducing gout flares during ULT initiation was confirmed in phase II and III trials (146, 147). Additionally, weekly subcutaneous administration of 160 mg rilonacept revealed no new safety signals, and its safety profile remained consistent with previous studies (148).

### 5.2 Targeting upstream metabolic signals

Beyond directly inhibiting the NLRP3 inflammasome, modulating the upstream metabolic signals regulating its activation also represents a promising strategy in gout management.

#### 5.2.1 Restoring uric acid homeostasis

Febuxostat and allopurinol, two xanthine oxidase (XOR) inhibitors, are foundational in the long-term management of gout. XOR plays a central role in purine catabolism by catalyzing the sequential oxidation of hypoxanthine to xanthine and then to uric acid (149). Febuxostat potently inhibits both the oxidase and dehydrogenase activities of XOR through high-affinity binding to the molybdenum-pterin active site. In contrast, allopurinol—a hypoxanthine analog—is metabolized to its active form, oxypurinol, which acts as a relatively weak competitive inhibitor at the same site (150, 151). By effectively inhibiting XOR, both agents reduce the production of uric acid.

Under normal physiological conditions, xanthine oxidoreductase (XOR) exists primarily in the dehydrogenase form, but during inflammation it converts to the oxidase form, catalyzing the production of reactive oxygen species (ROS) such as peroxynitrite. Febuxostat could reduce both mitoROS-dependent and mitoROS-independent IL-1β secretion by targeting XOR. It improves cellular bioenergetics—restoring intracellular ATP levels and mitochondrial function—through activation of the salvage pathway, thereby inhibiting NLRP3-driven IL-1β release and macrophage cell death (152). By inhibiting both enzymatic forms of XOR, febuxostat also attenuates oxidative stress and vascular inflammation, as demonstrated in models of renal ischemia-reperfusion injury where it reduced tissue damage via suppression of ROS (153, 154). These findings highlight the role of XOR in regulating inflammasome activation and support the therapeutic potential of XOR inhibition in inflammatory diseases.

#### 5.2.2 Modulating fatty acid synthesis

Fatty acid synthesis is implicated in immune processes such as B lymphocyte and human monocyte differentiation (155, 156). This metabolic pathway is tightly regulated by key enzymes, including fatty acid synthase (FASN) (157). Pharmacological inhibition of FASN with C75 has been shown to reduce reactive oxygen species (ROS) levels and suppress pyroptosis via activation of the Nrf2/HO-1 pathway. Concurrently, C75 treatment downregulates the expression of NLRP3, Caspase-1, IL-1β, and IL-18 (158), suggesting that FASN may act as a critical regulator of NLRP3 inflammasome activation in inflammatory diseases.

Dysregulated lipid metabolism, particularly increased synthesis of saturated fatty acids such as palmitate, can promote NLRP3 inflammasome activation by inducing mitochondrial dysfunction and endoplasmic reticulum stress (93). Pro-inflammatory macrophages depend mainly on glycolysis for energy, while alternatively activated macrophages acquire energy through fatty acid oxidation (FAO) (159). Notably, FAO also contributes to inflammasome activation in pro-inflammatory macrophages (160). Studies indicate that inhibiting FAO with trimetazidine reduces NLRP3 inflammasome activation, as shown by decreased cleavage of caspase-1 (161), suggesting a potential therapeutic strategy for inflammasome-related diseases.

ω-3 polyunsaturated fatty acids (ω-3 PUFAs) are well established for their lipid-lowering effects, particularly in treating hypertriglyceridemia. Beyond their role in lipid metabolism, ω-3 PUFAs also exhibit potent anti-inflammatory effects. For instance, they have been demonstrated to inhibit NLRP3 inflammasome activity in obese individuals by downregulating the expression of inflammasome-related genes in both adipocytes and macrophages (162). *In vitro* studies further support this mechanism, showing that treatment of macrophages with 20 μM ω-3 PUFAs effectively suppresses NLRP3 inflammasome activation, thereby inhibiting caspase-1 cleavage and reducing IL-1β secretion (95, 163). Additional *in vivo* evidence comes from murine GA model, wherein diets enriched with ω-3 PUFAs attenuated inflammation induced by subcutaneous injection of MSU crystals compared to standard diets—a finding corroborated by human case-control studies (164). Collectively, these findings suggest that modulation of fatty acid metabolism and inflammation by ω-3 PUFAs may represent a promising therapeutic strategy for conditions such as GA.

#### 5.2.3 Targeting lipid metabolism

As previously discussed, the accumulation of cholesterol crystals and oxidized lipids contributes significantly to NLRP3 inflammasome activation (72, 99), highlighting a promising therapeutic target for GA. Both oxLDL and cholesterol crystals are well-established NLRP3 agonists that trigger the canonical inflammasome pathway (100). Thus, compounds that attenuate oxLDL formation or cholesterol crystallization represent promising strategies for GA treatment.

Statins, known primarily for their lipid-lowering effects, also exhibit anti-inflammatory effects mediated through inhibition of the NLRP3 inflammasome. Given that many gout patients also require lipid-lowering therapy, the potential cardiovascular benefits of statins—including anti-inflammatory effects beyond lipid reduction—warrant particular interest (165). Studies have demonstrated that statins inhibit oxLDL- or TNF-α-induced NLRP3 inflammasome activation in vascular endothelial cells (166). Although statin use has not been broadly associated with reduced gout incidence, protective effects have been observed in patients receiving higher cumulative doses or longer treatment durations (167). Probucol, an anti-oxidative and lipid lowering drug molecule that has been shown to reduce serum low density lipoprotein-cholesterol in both preclinical and clinical studies (168). Recent research indicates that probucol administration significantly suppresses NLRP3 inflammasome activation, NF-κB signaling, and downstream proinflammatory cytokines including IL-1β, TNF-α, and IL-6 (169). Similarly, ezetimibe—an FDA-approved lipid-lowering drug—inhibits Niemann-Pick C1-Like 1-mediated cholesterol absorption at the intestinal brush border (170). It has also been found to attenuate NLRP3–IL-1β pathway activation in an autophagy-dependent mechanism (171).

Additionally, several natural products with lipid-modulating properties have demonstrated inhibitory effects on the NLRP3 inflammasome. For example, parthenolide directly targets caspase-1 to suppress inflammasome activity (172); glycyrrhizin exerts broad anti-inflammatory effects by inhibiting NLRP3 activation (173); and curcumin alleviates MSU-induced inflammation by suppressing IκBα degradation, NF-κB activation, mitochondrial dysfunction, and NLRP3 inflammasome activity (174).

#### 5.2.4 Regulating glycolysis

Glycolysis plays a critical regulatory role in the activation of the NLRP3 inflammasome (175). In macrophages, metabolic reprogramming toward glycolysis is a hallmark of inflammatory activation, and the switching of key glycolytic enzymes serves as a pivotal mechanism controlling NLRP3 inflammasome assembly and signaling. This glycolytic shift in immune cells represents a fundamental metabolic adaptation during inflammation.

Metformin, the only biguanide derivative in clinical use and a first-line therapy for type 2 diabetes mellitus (T2DM), exhibits anti-inflammatory properties that may benefit GA management. Its glucose-lowering effects involve multiple pathways, including suppression of hepatic gluconeogenesis (176), reduction of intestinal glucose absorption (177), and enhancement of peripheral glucose uptake and utilization (178). Notably, metformin has been shown to inhibit NLRP3 inflammasome activation via the AMPK/mTOR signaling pathway (179). Another glycolysis inhibitor, 2-deoxy-D-glucose (2-DG), also attenuates NLRP3 activation, potentially through alleviating oxidative stress (180).

Several small-molecule compounds are known to modulate glycolytic flux in macrophages and influence NLRP3 inflammasome activity. For instance, shikonin—a potent inhibitor of the pyruvate kinase M2 (PKM2) isoform (181)—suppresses the PKM2–EIF2AK2 pathway, thereby attenuating NLRP3 and AIM2 inflammasome activation and reducing the secretion of IL-1β, IL-18, and HMGB1 both *in vitro* and *in vivo* (182). Similarly, lactate production via anaerobic glycolysis contributes significantly to NLRP3 activation, and the lactate dehydrogenase A (LDHA) inhibitor GSK2837808A effectively suppressed NLRP3 activation and conferring protection in mouse models of MSU-induced peritonitis (183). Additionally, chaetocin, a histone methyltransferase inhibitor, has been shown to inhibit the expression of hypoxia-inducible factor-1α (HIF-1α) and hexokinase 2, the master regulators of glycolysis, and subsequently suppress NLRP3 inflammasome activation, leading to reduced IL-1β release in response to MSU stimulation (184).

Given the intricate relationship between the NLRP3 inflammasome and cellular glycolysis, these findings suggest that targeting both glycolysis and the NLRP3 inflammasome could represent a promising therapeutic strategy not only for gout but also for other inflammatory diseases.

## 6 Conclusion and future perspectives

This review provides an integrated metabolic-inflammatory framework that advances our understanding of acute GA pathogenesis. We establish that metabolic disturbances—including HUA, dyslipidemia, and glucose fluctuations—function as dual-action mediators in GA: they serve as the “priming soil” by upregulating NLRP3 inflammasome components and pro-IL-1β expression through NF-κB-dependent pathways, while simultaneously acting as the “ignition trigger” through direct inflammasome activation culminating in caspase-1 cleavage and mature IL-1β release. This “two-hit” model redefines acute gout flares not as passive consequences of crystal accumulation, but as active inflammatory processes driven by intricate crosstalk between systemic metabolic status and innate immune responses, thereby providing a comprehensive pathophysiological framework for understanding how metabolic signals lower the threshold for inflammatory activation.

A central innovation of this work is the systematic delineation of how individual metabolic factors engage specific NLRP3 activation pathways. These mechanisms reveal both shared principles —such as NF-κB-dependent priming—and unique effectors that distinguish each metabolic signal, including lysosomal rupture by cholesterol crystals and glycolytic reprogramming by glucose. Building upon this mechanistic insight, this review positions the metabolic milieu as an active participant in disease pathogenesis rather than a passive background condition. Collectively, these insights establish a rationale for a “dual-pathway” therapeutic approach that extends beyond gout to other metabolic-inflammatory disorders, positioning metabolic interventions as complementary strategies to conventional anti-inflammatory treatments.

Current therapeutic strategies, including nonsteroidal anti-inflammatory drugs, colchicine, and biologic agents targeting IL-1, primarily focus on suppressing inflammation. While effective in symptom control, these approaches do not address the metabolic signals. Therefore, future management of gout should adopt an integrated “dual-pathway” strategy: combining effective anti-inflammatory interventions with rigorous management of underlying metabolic abnormalities. Several promising research directions warrant further investigation: 1. Targeted Modulation of the NLRP3 Inflammasome: Developing highly efficient and safe specific NLRP3 inhibitors may offer novel therapeutic options for acute gout episodes, superior to conventional broad-spectrum anti-inflammatory treatments. 2. Elucidating Metabolic-Immune Crosstalk: In-depth exploration of how metabolites regulate the NLRP3 inflammasome signaling pathway may reveal new intervention targets. 3. Personalized Treatment and Prevention Strategies: Building predictive models based on individual metabolic and immune profiles could facilitate early identification of high-risk individuals and enable personalized metabolic interventions, shifting the paradigm from treating acute attacks to preventing their occurrence.

In conclusion, a deeper understanding of the interaction between metabolic signals and the NLRP3 inflammasome not only enhances our insight into the pathogenesis of gout but also paves the way for innovative therapeutic strategies and fundamental prevention approaches, ultimately improving clinical outcomes for patients.
